# Review-Microwave Radar Sensing Systems for Search and Rescue Purposes

**DOI:** 10.3390/s19132879

**Published:** 2019-06-28

**Authors:** Nguyen Thi Phuoc Van, Liqiong Tang, Veysel Demir, Syed Faraz Hasan, Nguyen Duc Minh, Subhas Mukhopadhyay

**Affiliations:** 1 Department of Mechanical and Electrical Engineering, SFAT., Massey University, Manawatu Private Bag 11 222, Palmerston North 4442, New Zealand; 2 Electronics Faculty, Hanoi University of Industry, Number 298, Cau Dien Street, Bac Tu Liem District, Ha Noi 100000, Vietnam; 3 Electrical Engineering Department, College of Engineering and Engineering Technology, Northern Illinois University, DeKalb, IL 60115-2854, USA; 4 School of Electronics and Telecommunications, Hanoi University of Science and Technology, Hanoi 100000, Vietnam; 5 School of Engineering, MQ Centre for Smart Green Cities, Macquarie University, New South Wales, NSW 2109, Australia

**Keywords:** vital signals, finding survivors, Doppler radar, heartbeat, breathing signal, detection probability

## Abstract

This paper presents a survey of recent developments using Doppler radar sensor in searching and locating an alive person under debris or behind a wall. Locating a human and detecting the vital signs such as breathing rate and heartbeat using a microwave sensor is a non-invasive technique. Recently, many hardware structures, signal processing approaches, and integrated systems have been introduced by researchers in this field. The purpose is to enhance the accuracy of vital signs’ detection and location detection and reduce energy consumption. This work concentrates on the representative research on sensing systems that can find alive people under rubble when an earthquake or other disasters occur. In this paper, various operating principles and system architectures for finding survivors using the microwave radar sensors are reviewed. A comparison between these systems is also discussed.

## 1. Introduction

The non-invasive detection ability of radar sensor systems has recently gained much attention among researchers. This ability leads to many interesting applications of the microwave sensing systems, such as healthcare monitoring, defense, security, smart homes, and alive person search and rescue. The Doppler radar sensor system can be used to detect and monitor movement of human tissues and organs without touching the body and provides a non-invasive technique to diagnose diseases related to heart, lungs, and the vascular system [1]. The first vital signs’ sensing system was introduced by Caro et al. in [2]. This system was used to determine the apnea phenomenon of infants, in which an alarm was turned on when the apnea lasted more than 30 s. Their recommended distance from human to antennae was around 50 cm. After that, many studies tried to improve the quality of the vital signs’ sensing system by improving hardware structures and signal processing techniques [3,4,5,6,7,8,9,10,11,12,13,14,15,16,17,18,19,20,21,22,23,24,25,26,27,28,29,30,31,32,33,34,35,36,37,38,39,40,41,42,43,44].

In [5], Lee and Lin proposed an arterial pulse wave analyzer based on a CW Doppler microwave, which operated at a frequency of 24.125 GHz. Their system could detect the movement of the arterial wall accurately. In 1990 and 1996, two patents [10,14] were registered in the U.S.A Both of them focused on the medical application for the radar sensor. In a radar sensing system, the antennae play an important role in increasing the sensing ability. The works in [28,32,35,44,45,46,47,48,49] focused on improving the sensitivity of the antennae system in order to detect cancer tumors or thorax movements. Hall et al. [42] presented a survey on an intelligent phase-array CW sensor system for monitoring vital signs continuously. In recent years, non-contact radar sensors have been combined with artificial intelligence (AI) to predict clinical events [43,50]. Research in this field suggests a high potential of smart radar sensors.

To improve the quality of vital signs’ detection, many researchers focused on signal processing methods to achieve a high accuracy of the breathing rate and heartbeat [51,52,53,54,55,56,57,58,59,60,61,62,63,64,65,66,67,68,69,70,71,72] or building models to estimate the accuracy of these systems [73,74]. The work in [58] presented the maximum likelihood estimator (MLE) and generalized likelihood ratio test (GLRT) techniques that were used to estimate the breathing rate and heartbeat. Their signal processing showed the ability to separate multiple vital signals. The attempts to calibrate for the signal spectrum and to remove the breathing harmonics were discussed in [59,63]. Additionally, in [63], the clutter suppression of random body movements was proposed to increase the quality of vital signs’ signal detection. Similarly, Gu et al. [60] investigated a random body movement cancellation (RBMC) technique by combining a camera system with the microwave sensor. The random body movements recorded by the camera were used to compensate the phase shift in the radar sensor. Another approach to enhance the breathing rate and heartbeat detection in the radar sensor is cyclostationary [62,70]. The author in [62,70] reported that the result of extracting signals depended less on signal-to-noise ratio (SNR) or filtering technique. When the radar sensor system had to detect vital signs through obstacles, more effort needs to be made on signal processing to extract the desired signals. Yan [68] and Liang [69] introduced their solutions to de-noise and detect vital signs’ signal through a wall.

Besides the signal processing techniques for vital signs’ detection of a radar sensor system, the hardware development is crucial to make these systems more reliable. The development of semi-conductor technology has conferred many advantages to the radar system. The size of the radar system is significantly reduced. The radar sensor is now integrated with other circuits (signal processing circuit, ADC, control circuits, and so on) on the same board. Many studies have paid attention to making the radar sensor even smaller, saving power and increasing its sensitivity [75,76,77,78,79,80,81], as well as detecting distance or direction [82,83,84,85,86,87,88,89,90,91,92,93,94]. Most on-chip radar sensor systems operate at quite high frequencies (above 5 GHz). This research has made contributions to various applications in daily life such as observing the respiratory/sleeping patterns of humans [95,96], diagnosing respiratory diseases [97,98], collecting cardiac/respiration of rats [99], and monitoring the heartbeat of cows [100] in a dairy farm.

Another important application of the Doppler radar sensor is to search for and locate the position of victims under the rubble of collapsed buildings. Owing to the obstacles between the living victim and the radar sensor, the penetration property of the microwave radar sensor for such an application is an important factor to consider. The penetration ability of a microwave sensor system depends on the operating frequency and transmitting power. Therefore, several studies chose the *L* and *S* band as the operating frequencies for their system [82,92]. This paper reviews this crucial application of the radar sensor. Based on two general types of radar sensors, continuous wave (CW) and ultra-wideband (UWB) systems, the representative radar sensor systems for the purpose of research and rescue are reported in this work. First, the background research and literature review regarding radar sensors are presented. Then, various types of radar sensors for search and rescue purposes are discussed from the operating principles to hardware structures. Finally, the conclusion and future perspectives are presented.

## 2. Background Research and Literature Review

### 2.1. Hardware Developments

**Figure 1:**
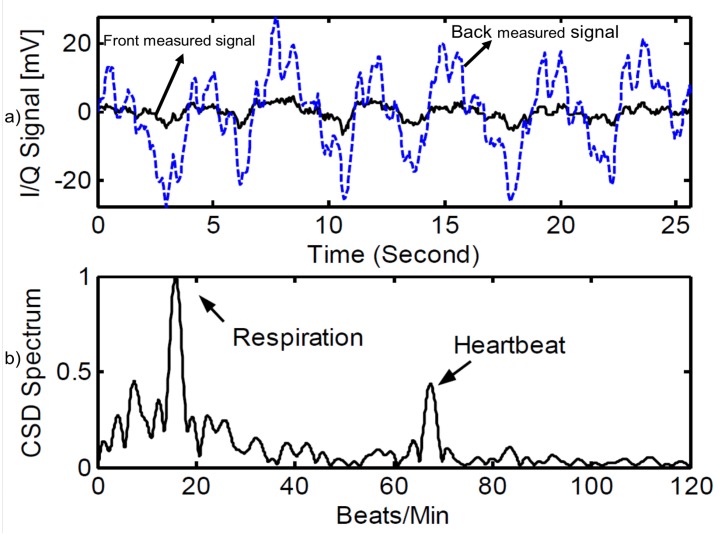
Received signal in the time domain (**a**) and in the frequency domain (**b**) at a distance of 0.5 m [101].

In the last five decades, the improvement of semi-conductor technology, embedded computing, and artificial intelligence (AI) techniques has enabled significant improvement in the vital sign detection Doppler sensor systems. The cumbersome microwave radar sensor of the past has been replaced by smaller, smarter, and higher sensitivity systems. An early respiratory monitoring system [4] was attached to an X-ray and triggered this machine automatically by instance of respiration. This system is quite simple. It consists of an oscillator, quadrature mixer, direction detector circuit, differentiators, and a trigger circuit. Another CW microwave radar for vital signs’ detection was discussed in [8] with a size of 20 cm × 28 cm × 18 cm and large attached antennae. Later, the blooming of semi-conductor technology helped to reduce the size of the vital sign-detecting radar sensor system. For example, the 5.8-GHz radar sensor receiver chip of 0.13-μm CMOS [101] is as small as 1.2 mm × 1.2 mm. This system can detect the breathing rate of humans at the distance of 1.5 m. The detection results of breathing rate and heartbeat when a person sits at a distance of 0.5 m are displayed in Figure 1. The respiration and heartbeat can be found easily from the spectrum of the received signal.

**Figure 2:**
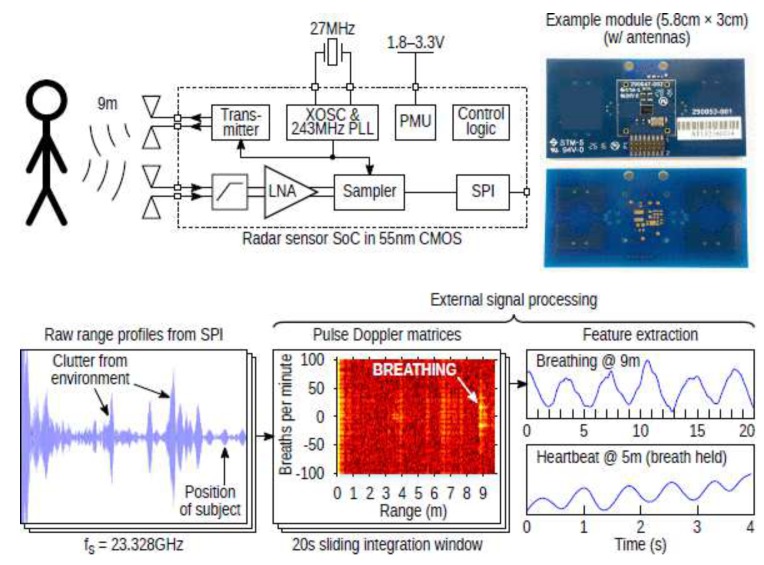
System overview and real-life application example with measured performance [102]. LNA, linear amplifier.

The system on chip (SoC) radar sensor has become very popular as it has many applications. The work in [102] introduced an SoC radar sensor with RF interference rejection and integration of power management clock generation functions. To operate this system, the external antennae and crystal need to be connected within this compact kit. The block diagram, the example module, and application results of this system are illustrated in Figure 2. The example module had a size of 5.8 cm × 3 cm, and it could detect the breathing rate at a distance of 9 m and heartbeat at a distance of 5 m. When this system operates in a condensed environment, the detection distance might be reduced, but it still has high potential for search and rescue applications.

The development in hardware of the microwave radar sensor goes along with processing techniques to improve the accuracy of vital signs’ detection. The next section discusses the different algorithms and methods to process signals of the microwave radar sensors.

### 2.2. Signal Processing Techniques

There are many techniques to obtain and classify the breathing rate/heartbeat from the baseband received signal of the microwave sensor. The fast Fourier transform (FFT) and continuous wavelet transform (CWT) [64,65,66,67,68,69,70,71,72,103,104,105,106] are considered as very basic methods to retrieve the respiratory rate. These techniques can find the frequency spectrum of received signals, based on the peak of the spectrum in a specific frequency range, and the breathing rate or heartbeat can be estimated. However, the microwave radar sensor operates in a noisy environment with a variety of noises such as random body movements, flicker noise, clutter noise, leakage noises between transmitting antennae and receiving antenna, and so on. Therefore, in many application, simply applying FFT or CWT on the received signal is not sufficient to obtain the desired signals (vital signs).

Another popular technique to find the pattern in the data is principal component analysis (PCA). In this method, principal components of vector data can be found by forming the orthogonal matrix in which rows are eigenvectors of the covariance matrix of vector data [107]. This method was then combined with WT or short time Fourier transform (STFT) to process multivariate statistical data [108]. This signal processing method can extract the respiratory rate from the pulse oximeter’s photoplethysmographic signals with high accuracy.

In the received signal of the radar sensor, the breathing signal has higher energy than the heartbeat signal. To reduce the effect of breathing rate harmonics on the heartbeat signal, the chest displacement signal is firstly broken down into intrinsic components. The breathing rate and heartbeat can be restructured from those components in the time domain. This technique is called empirical mode decomposition (EMD) [109]. After applying the EMD on the received signal, the simple FFT can be applied to find the breathing rate and heartbeat.

To reduce the random body movement, Li et al. [30,110] proposed a method that detects signals from both sides of a human. These signals are combined to cancel out the noise caused by random body movement, then the arctangent or complex signal demodulations are applied before using FFT to find the breathing rate and heartbeat. Similarly, the work in [63] also used two receiving antennae to detect the vital signs. However, they just simply chose the strong signal to process. Then, the moving filter was applied to the selected signal to remove the quasi-static clutter.

Another technique to reduce the flicker noise was mentioned in [61] by using the harmonic radar. This work proposed a harmonic CW radar sensing system that operated at two frequencies (fundamental and harmonic). They reported that in their system, the flicker noise was reduced significantly, while SNR was increased.

Recently, Yu et al. [70] proposed an approach based on higher order cyclostationarity to detect the heartbeat and respiration of a person. The third-order cyclic cumulant was applied to detect the vital signs. This method could reduce the harmonic interferences, random body movements, and clutter noise. It also allowed the radar sensor to detect the weak signals with low SNR values.

**Figure 3:**
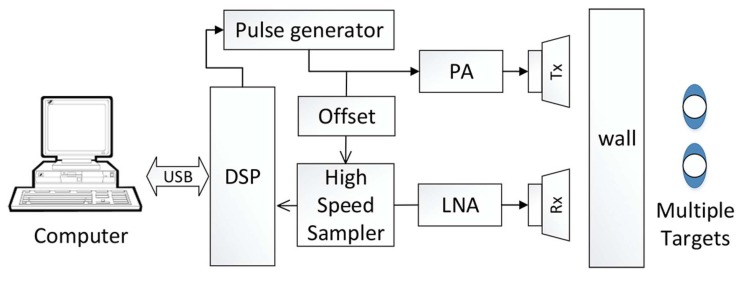
Block diagram to test multiple targets’ detection [68]. PA, power amplifier.

**Figure 4:**
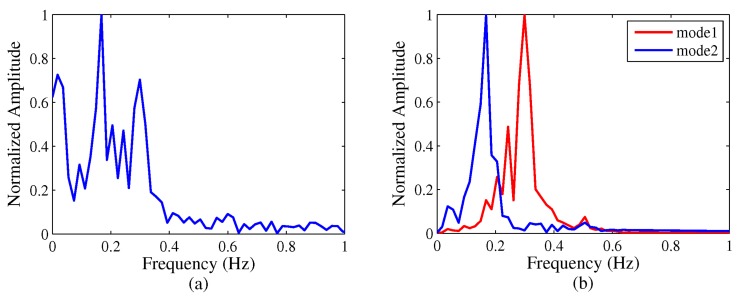
Traditional FFT method (**a**) versus the variational mode decomposition (VMD)-based method (**b**) [68].

Besides the vital signs’ detection techniques, target tracking based on the radar sensor is also very important in search and rescue or defense applications. Yan et al. [68] proposed an approach based on an algorithm called variational mode decomposition (VMD) to track different targets behind a wall. The setup of the testing system is shown in Figure 3. This method decomposed the breathing signals into various sub-signals. The separated signals can be tracked by the VMD algorithm. The tracking algorithm consisted of four steps: determine traversed range bins, VMD algorithm, breathing recognition, and Hilbert transform. The result of this proposed algorithm is compared with the conventional FFT in Figure 4. It is obvious that the VMD algorithm approach is a good choice in the case of multiple target detection based on a microwave radar sensor.

Another powerful algorithm to detect breathing rates of multiple subjects is the independent component analysis (ICA) [111,112] technique. This method allows blind segregation of data into independent sources. This method was applied to separate the breathing rates of three people successfully by an step frequency continuous wave (SFCW) radar sensor [111].

### 2.3. Problems and Solutions in the Search and Rescue Scenario

There are several issues that one has to deal with when developing a microwave radar sensor to detect alive persons under ruble. First, the null point problem in the microwave radar sensor appears when the phase shift due to the distance from the target to the sensor is an even multiple of $\frac{\pi }{2}$. In this case, the received signal approximates a zero value. To overcome this problem, the I/Q microwave radar is a good solution. Droitcour et al. [113] showed in their work that the null points issue can be reduced by using a quadrature receiver in the radar sensor system. Moreover, other methods that can be used to get rid of this problem are double-sideband transmission [86], complex signal demodulation [110], and arctangent demodulation [114].

Secondly, the vital signs’ detection quality of the microwave radar sensor is effected negatively by the motion artifacts’ noise or multiple subjects’ interference. Multiple-input, multiple-output (MIMO) or single-input, multiple-output (SIMO) methods could be used to alleviate these problems [47,115,116]. The SIMO/MIMO antennae system was combined with the SIMO/MIMO signal processing technique to differentiate between multiple subjects. Moreover, the random body movement and motion artifacts’ noises can be canceled by using two detecting transceivers [116]. To enhance the detecting of multiple targets and the sensitivity of the radar sensing system, a hybrid system that has two operation modes was proposed. In this system, Doppler mode was used to detect the vital signs, and frequency modulation continuous wave (FMCW) mode took the responsibility to find the absolute range of the target [117].

Other obvious interferences that the microwave search and rescue sensor has to deal with are the interference caused by an operator around the radar antennae and the noise in the shadowing area. The work in [118] introduced a dual-frequency CW radar operated at 5.57- and 35-GHz frequencies to suppress the interference from the radar operator. The radar sensor consisted of two transceivers operated at two frequencies at the same time. The lower frequency signal of this system can penetrate through ruble, while the higher frequency signal cannot go too far and is used to detect the vital signs of the operator. The receiving signal at the higher frequency transceiver was used to remove the operator interference in the lower frequency part. This system showed high potential application for search and rescue purposes because it showed efficient suppression of interference from the operator. Going along with the hardware development to enhance the quality of the radar sensing system is the signal processing algorithm. The shadowing effect was diminished by applying a proposed algorithm based on wavelet entropy in a bistatic UWB radar [119]. In this algorithm, the difference between periodic respiration and random noise and the entropy of the human target were utilized to detect two people at the same time. One person was closer to the radar, and one person was in the shadowing region. This method can be combined with the multiple channel system to detect multiple subjects under debris/ruble at the same time.

## 3. Classification of Radar Sensors Based on Transmitting Wave Forms

### 3.1. Single-Tone CW Radar Sensor

**Figure 5:**
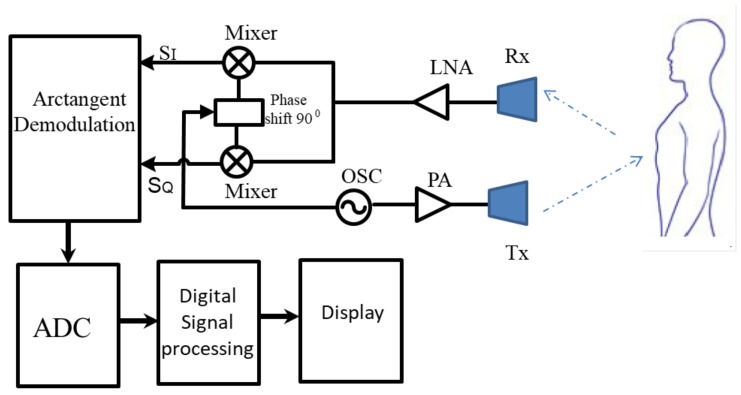
Continuous wave system diagram.

A typical functional block diagram of the CW radar sensor system is shown in Figure 5. The system consists of an oscillator (OSC), I/Q demodulator, arctangent demodulator, analog-to-digital converter (ADC), and a digital signal processing and display unit. The OSC is the microwave signal source, and this signal is amplified by a power amplifier (PA) before being sent towards the human position through the transmitting antenna Tx. The receiving antenna Rx of the sensor captures the reflected signal from the human. This signal is amplified by a linear amplifier (LNA). After that, the signal is downconverted by a quadrature modulator to create two orthogonal signals, $S_{I}$ and $S_{Q}$. Two orthogonal signals are used as the inputs for the arctangent demodulator. The analog signal from the arctangent modulation unit is digitalized by the ADC before sending to the DSP to extract useful information. The results (vital signs such as breathing rate, heartbeat, and so on) are shown on the display unit.

The generated signal of the CW radar sensor system can be represented by:

(1) $$\begin{array}{c} V_{T}\left(t\right)=A_{T}\cos\left(2\pi f_{0}t+\phi \left(t\right)\right) \end{array}$$

where $A_{T}$ is the amplitude of the transmitted signal, $f_{0}$ is the carrier frequency, *t* is the time, and $\phi \left(t\right)$ is the phase noise. The signal $V_{T}\left(t\right)$ is then reflected by the human’s chest. The chest displacement can be considered as a periodic signal $l\left(t\right)$. At the receiver, this signal is downconverted by the I/Q demodulator. The purpose of using I/Q demodulation is to alleviate the null-points problem [41]. The base band signal at the output of demodulator can be written as:

(2) $$\begin{array}{c} B_{O}\left(t\right)\approx arctan\left(\theta_{0}+\frac{4\pi l(t)}{\lambda }+▵\theta \left(t\right)\right) \end{array}$$

The thorax displacement l(t) is much smaller than the value λ; therefore, the small angle rule can be used in Equation (2). The output signal of the radar sensor system is proportional to the chest movement.

(3) $$\begin{array}{c} B_{O}\left(t\right)\approx \theta_{0}+\frac{4\pi l(t)}{\lambda }+▵\theta \left(t\right) \end{array}$$

where $\theta_{0}$ is the phase shift due to the distance from the human location to the antennae system and ▵θ(t) is the phase noise.

#### CW Radar Topologies

**Figure 6:**
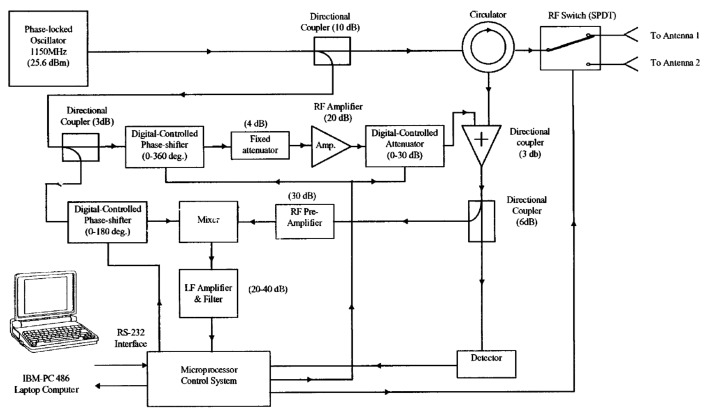
Schematic of the 1.15-GHz microwave radar [82].

In 2000, Kum-Mu Chen et al. [82] constructed two CW microwave life-detection systems, which operated at 450 MHz and 1.15 GHz, respectively. Their experiment results showed that the 1.15-GHz microwave radar could penetrate deeper though the ruble with metallic wire. The block diagram of this life-detector is shown in Figure 6. A stable CW signal (power 25.6 dBm) was generated by a phase-locked oscillator. This signal went through a 10-dB directional coupler and a circulator before reaching the RF switch to the antenna. A 10-dB directional coupler divided the signal from OSC into two parts: 90% of the power goes to the circulator, and 10% goes to the 3-dB directional coupler. At the second directional coupler, the 40-mW power is split into two equal parts, which are used as a local reference for the mixer and to drive the clutter cancellation circuit. In this system, the two antennae are stimulated sequentially. The signals from both antennae are associated to decrease the background noise. The clutter noise is canceled by the combination of the digitally-controlled phase-shifter, fixed attenuator, amplifier, and digitally-controlled attenuator. In addition, the system sensitivity is increased by a phase-shifter connected to the local port of the mixer [82].

**Figure 7:**
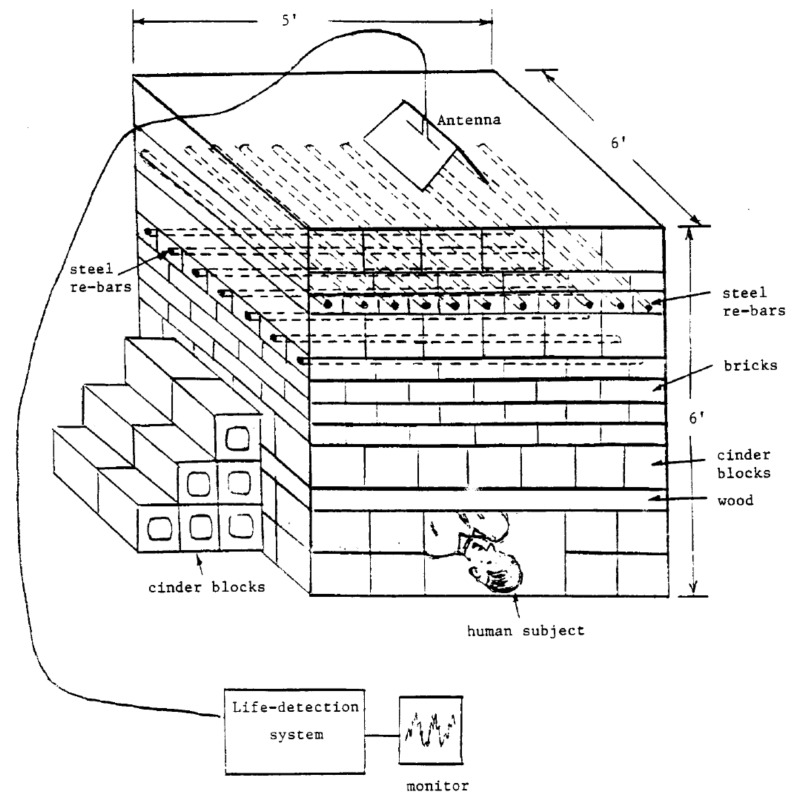
Testing performance of Kum-Mu Chen et al.’s [82] system on the earthquake rubble model of Michigan State University.

This system was tested on the simulated earthquake rubble at the Electromagnetics Laboratory, Michigan State University (Figure 7). The system can detect the breathing rate and heartbeat from a distance of seven feet including three feet of rubble with metallic wire mesh. This system showed a good result with the dual antennae system, which helped to reduce the background noise and the noise created by the nearby moving operators.

**Figure 8:**
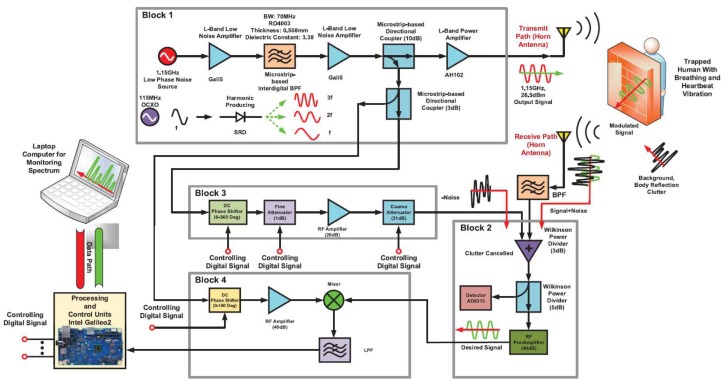
Schematic of the life-detector system [92].

Another system was developed by Jalai Bidgoli et al. [92] with the same purpose of finding survivors. This system operated at the same frequency of [82] (1.15 GHz) with a similar transmitting power (26.5 dBm). Their system had the ability to cancel the clutter and other noise sources, in which a microprocessor-based system was used to eliminate clutter. The computer was connected to the system to control and monitor the reflected signal. Two horn antennae were used in this work. The schematic presentation of their proposed system is shown in Figure 8. There were four main blocks in this system. The first block generated a transmitting signal; Blocks 2 and 3 took the responsibility of noise cancellation; while Block 4 downconverted the RF signal to the baseband signal. They reported that this system can detect the breathing rate at a 10-m thickness with standard density materials.

### 3.2. Frequency Modulation Continuous Wave Radar Sensor

**Figure 9:**
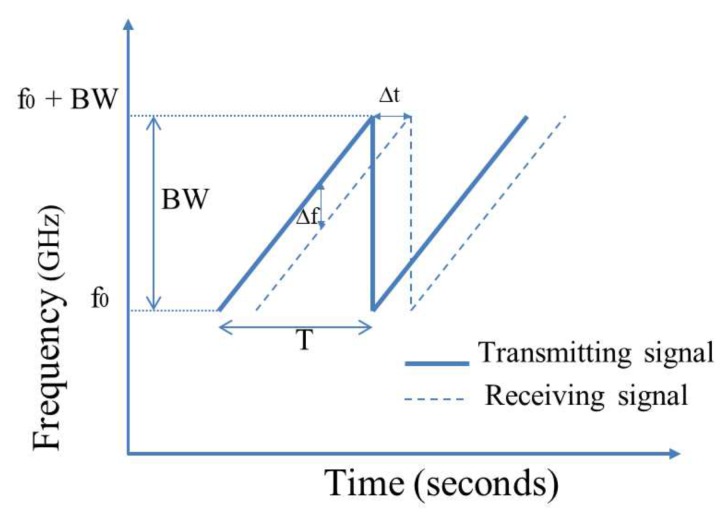
Frequency versus time of transmitting and receiving signal at frequency modulation continuous wave (FMCW) radar. BW, bandwidth.

The block diagram of the FMCW radar sensor is similar to the CW sensor, as shown in Figure 5, but the OSC should be replaced by a voltage control oscillator (VCO). The diagram of frequency versus time of FMCW radar is displayed in Figure 9, where BW is the bandwidth of the transmitting signal, ▵f is beat frequency, and ▵t is the time delay.

In the FMCW radar system, the transmitter sends a linear frequency signal from $f_{0}$–$f_{0}+B$ toward the person’s position. Owing to the person’s movement and the distance between the person and the radar sensor, the received signal is shifted in the frequency and time domain, as illustrated in Figure 5. The distance from the target to the radar sensor and the range resolution can be estimated as [41,120]:

(4) $$\begin{array}{c} d=\frac{cT▵f}{2BW} \end{array}$$

(5) $$\begin{array}{c} ▵d=\frac{c}{2BW} \end{array}$$

where BW is the bandwidth of the transmitted signal, ▵f is beat frequency, and ▵t is the time delay. *c* is the speed of light, and *T* is the pulse repetition period of the transmitted signal.

**Figure 10:**
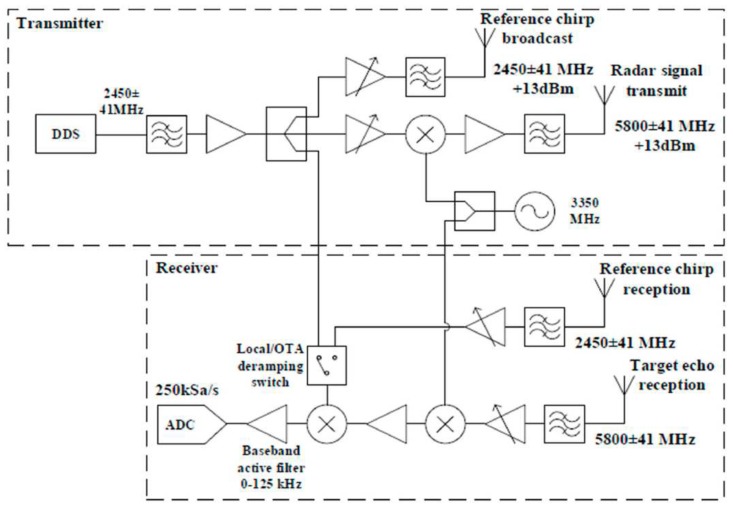
Topology of the FMCW radar used to detect humans through a wall [121].

In the work of [121], they applied the singular value decomposition (SVD) algorithm on the obtained signal from a so-called Soprano FMCW system. The hardware schematic is shown in Figure 10. This system was first developed by Chen et al. [122]. The central operating frequency of the sensor was 5.8 GHz with a bandwidth of 83.5 MHz. The transmitting power was 13 dBm, and the antenna gain was 12 dBm. This system was setup outside a residential house with a 33-cm brick wall to detect people inside the living room (2.8 m × 4.7 m).

**Figure 11:**
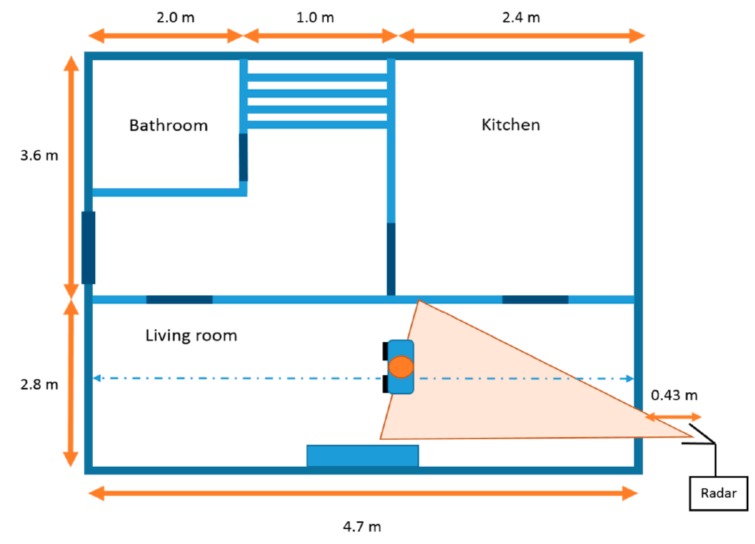
Experiment setup of the FMCW radar to detect a human through a wall [121].

The measurement setup is shown in Figure 11. The received signals in this radar system were processed by singular value decomposition (SVD) and moving target indicator (MTI) techniques to the detect location and the moving trend of the subject during the measurement.

### 3.3. Hybrid FMCW-CW Radar Sensor

**Figure 12:**
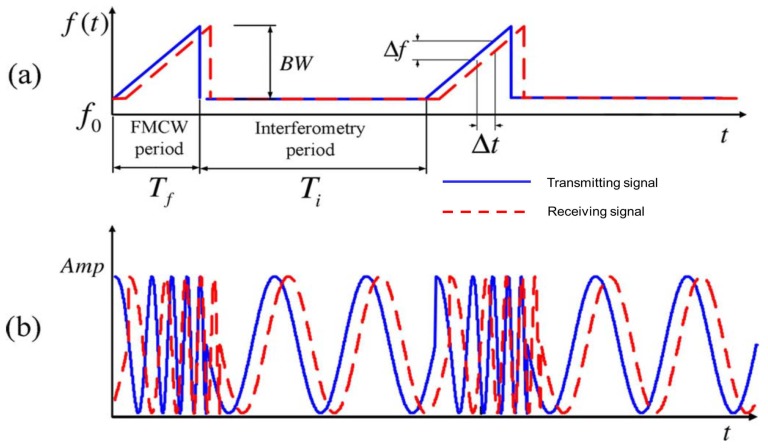
Transmitting and receiving signal of the FMCW-CW radar in the (**a**) frequency domain and (**b**) time domain [123].

To combine the advantages of range detection in FMCW radar and small displacement detection in the single-tone CW radar sensor, Guochao Wang et al. [123] introduced a hybrid system that operates at FMCW and CW alternatively to obtain the range and vital signs of a human. The transmitted and received signals in the frequency and time domains are demonstrated in Figure 12. The FMCW period provides the distance information, while the thorax displacements can be obtained from CW mode.

Both the works in [82] and [92] introduced a life-detector with high penetration capability. Based on their high directional antennae system, the direction of the victim under the debris might be found, but the distance from human to the radar antennae cannot be estimated. In these studies, they did not mention the range detection ability of their system. To enable both functions of range and vital signs’ detection, the hybrid FMCW-CW microwave radar seems to be a good choice [123].

### 3.4. Stepped-Frequency Continuous Wave Radar

**Figure 13:**
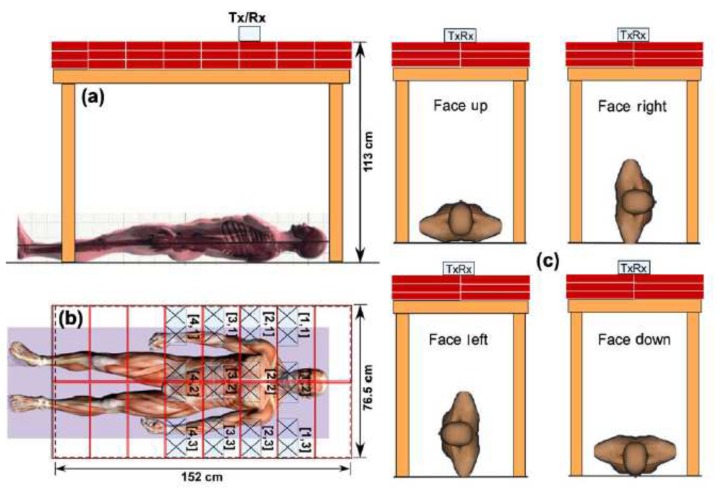
Experiment setup of the step frequency continuous wave (SFCW) radar to detect a human through brick: (**a**) size view, (**b**) top view, and (**c**) postures [124].

The SFCW has many advantages such as flexible frequency control and high dynamic range, and it can be easily calibrated at different frequencies [124,125]. The work in [124] performed some physical experiments that could simulate finding alive people under debris. The measurement setup of this work is described in Figure 13. The measured subject was asked to lie under bricks on the floor as in Figure 13a. The thickness of the brick layer was changed from 0–20 cm. The subject was asked to perform four postures as in Figure 13c. A total of 201 frequency steps from 300–1300 MHz were setup. The combination of the vector network analyzer (VNA) Model E5061A, two antennae, and a PC was utilized as the SFCW system.

**Figure 14:**
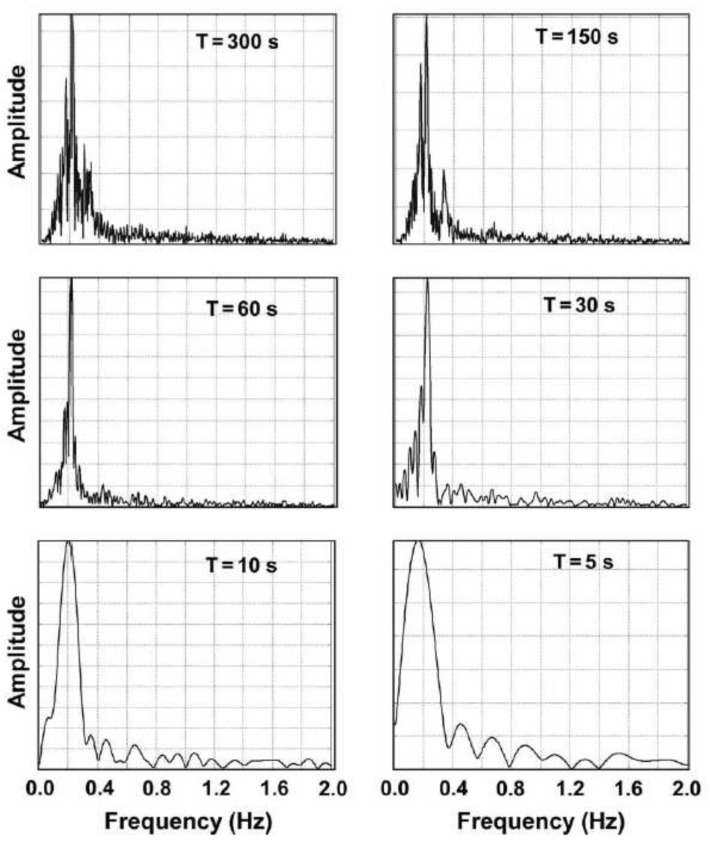
Breathing rate of a person in a face up posture at different time resolutions [124].

This work showed very encouraging results for human rescue application. The breathing rate of a human can be detected through the obstacle, and the posture of the subject is not critical. The measuring time to find the respiratory rate of a person is quite small (5 s), as shown in Figure 14. For search and rescue purposes, the breathing rate should be considered as the main signal to find the live subject. An alternative option to detect both the location and respiratory rate of person is to use pulse-based radar sensor systems. These types of radars are discussed in the next sections.

### 3.5. Random Noise UWB Radar Sensor

Another promising UWB radar is the random noise or random signal UWB radar. Its operating principle is similar to the short-pulse UWB sensor, but the short-pulse generator block is replaced by a band-limited random noise generator. This remote sensing system has high range and velocity resolutions. The effect of the antennae leakage and the radio frequency interference in this system is eliminated by its own waveform signal [126,127].

**Figure 15:**
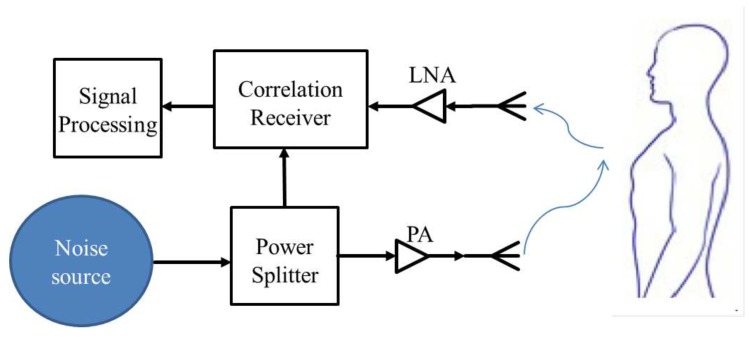
Diagram of a basic random noise UWB radar sensor.

For searching and locating people under debris when human-caused/natural disasters occur, the UWB radar is a good choice since it also can detect the vital signs and the location of the target. In 2004, Narayanan et al. [128] introduced the basic block diagram of random noise UWB radar as described in Figure 15. This sensing system can detect a trihedral reflector behind a wall.

**Figure 16:**
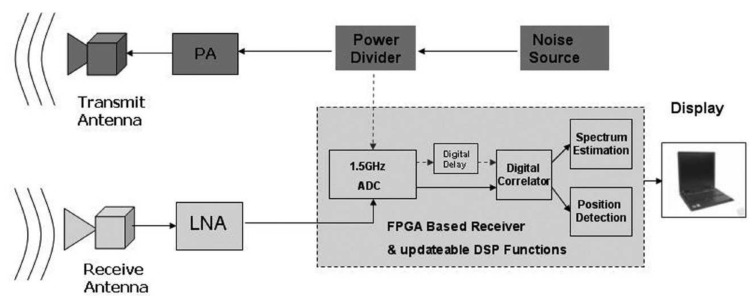
A schematic of a digital random noise UWB radar sensor [127].

Later, in 2010, Chieh-ping Lai et al. [127] proposed a digital UWB random noise radar sensor system for search and rescue purposes. The topology of the system is shown in Figure 16. In this design, an FPGA-based receiver was developed. The operating bandwidth of the system from 350 MHz–750 MHz corresponds to 37.5-cm resolution. In this work, the chip-based generator was used as the source noise, and the software-defined technique was utilized to reduce the size and power consumption of the radar system.

**Figure 17:**
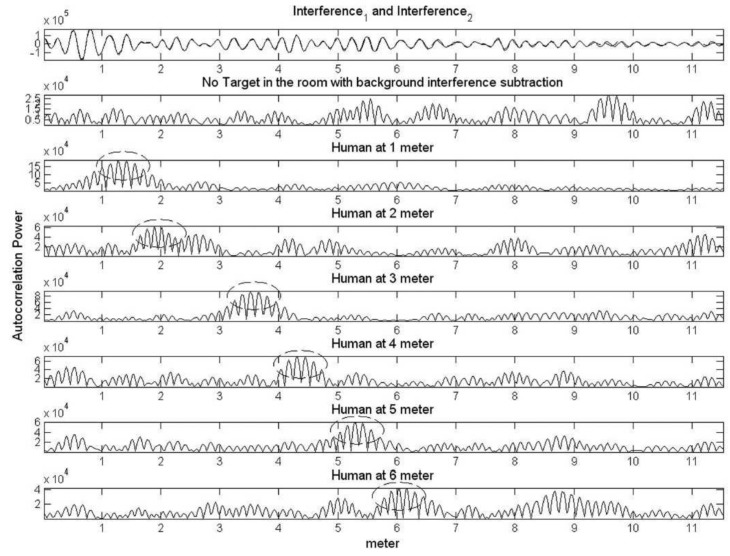
Detecting human movement through a wall using a digital random noise UWB radar sensor [127].

The results of Chieh-Ping Lai’s human detection system is presented in Figure 17. From this figure, we can see that this radar system can locate the human position at a distance of 6 m through the wall.

### 3.6. Pulse-Based Radar Sensors

**Figure 18:**
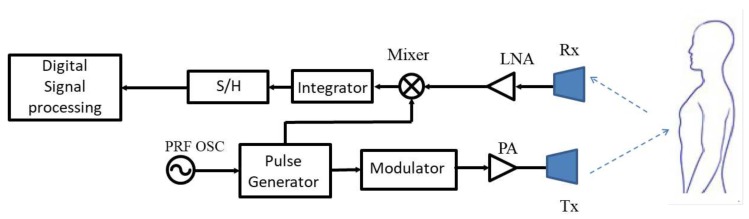
Block diagram of a typical UWB radar system. PRF, pulse repeat frequency; OSC, oscillator.

A pulse-based UWB radar has two operating modes: emitting and silence. A short pulse is sent in emitting mode, and the echo signal returns to the radar in silent mode. The range and velocity of the target can be detected by comparing the transmitted pulse and the received pulse. A basic block diagram of an UWB microwave radar sensor is shown in Figure 18. The desired waveform is created by the pulse generator. The pulse repeat frequency (PRF) is determined by a local oscillator. The pulse signal is modulated by a modulator before being amplified and emitted through the transmitting antenna ($T_{x}$). The echo signal is amplified by a linear amplifier before being downconverted to the baseband.

The transmitting signal ($s_{i}\left(t\right)$) in the time domain for the ith frame can be written as [129]:

(6) $$\begin{array}{c} s_{i}\left(t\right)=p\left(t-iT_{f}\right)cos\left(2\pi f_{0}\left(t-iT_{f}\right)\right) \end{array}$$

where $f_{0}$ is the carrier frequency, $p(t-iT_{f})$ is the impulse signal, and $T_{f}$ is the duration of the frame ($T_{f}$=$\frac{1}{f_{p}}$). $f_{p}$ is the pulse repetition frequency. The received signal after being amplified by LNA is given as follows.

(7) $$\begin{array}{c} r_{i}\left(t\right)=Ap\left[t-iT_{f}-\tau \left(t\right)\right]cos\left[2\pi f_{c}\left(t-iT_{f}-\tau \left(t\right)\right)\right] \end{array}$$

where *A* is the amplitude of the signal and depends on the gain of LNA and the transmission loss due to the environment between the radar and target. τ(t) is the propagation delay, equal to the round-trip time of a radar pulse. The range to the target (R(t)) can be estimated from the propagation delay τ(t) as:

(8) $$\begin{array}{c} R\left(t\right)=\frac{c\tau (t)}{2} \end{array}$$

The thorax movement of a human can be obtained by performing the Fourier transform on the received signal.

**Figure 19:**
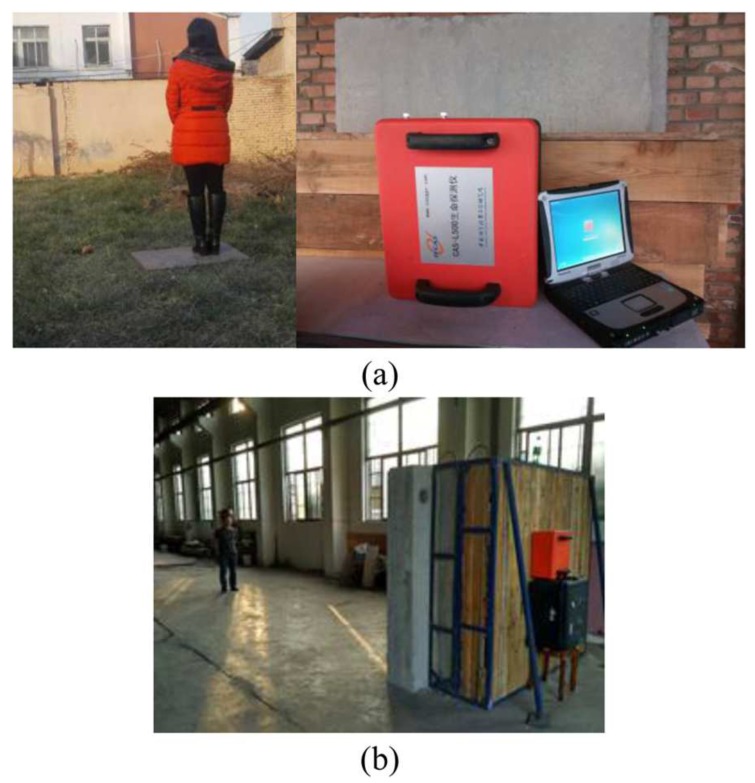
Vital sign detection experiments for a human subject by the UWB radar sensor: (**a**) outdoors and (**b**) indoors [94].

To improve the performance of the short pulse radar, a coding technique is utilized to increase resolution and avoid the multi-user interference. The transmitted signal is coded by pseudo-noise code in the transmitting block. This radar is a so-called as pseudo-random UWB radar to be used to detect the vital signs of humans [130,131,132]. To enhance the penetration property in the pulse-based UWB radar, the transmitted power and the carrier frequency of the system should be considered carefully. Moreover, the signal processing method is more challenging in such a dense transmitting environment. The works in [69] and [94] reported a de-noising method for detection of through-wall vital signs based on the UWB radar sensor. The experimental setup is shown in Figure 19. The thickness of the wall was 1 m, and the human target was measured at varying distances from 3 m–12 m.

The parameters of the UWB radar are given in Table 1.

**Figure 20:**
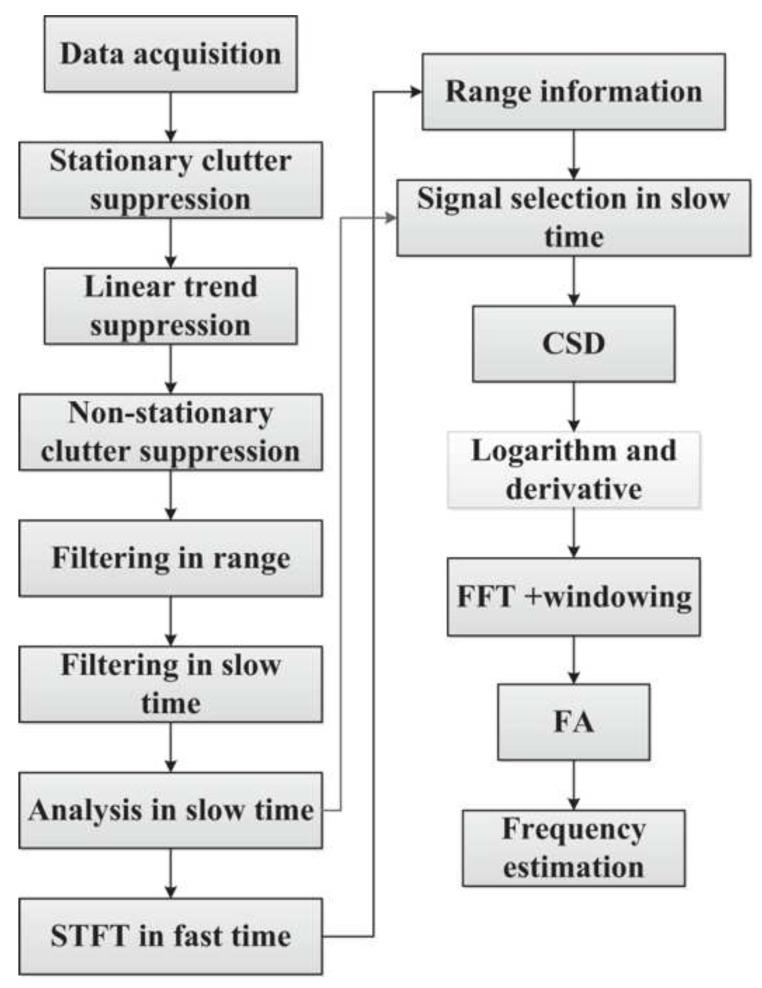
Detection algorithm [94].

There are two important points to be noted from Table 1. First, the center frequency of this system is quite low (400 MHz). Second, the amplitude of the transmitted signal is quite high (50 V). Both conditions, low operating frequency and high power, make sure that the signal can penetrate through the dense rubble to find live victims when there is a disaster. Moreover, Liang [94] also proposed a detection algorithm to provide range information and vital signs like breathing rate and heartbeat. The flowchart is displayed in Figure 20. Several de-noising techniques were applied: First, the stationary clutter, linear trend, and non-stationary clutter were suppressed. After that, the signal was filtered in range and in slow time. The next step was to analyze in slow time, then input this signal to FFT and window to find the frequency. The output signal of STFT was used for range information.

**Figure 21:**
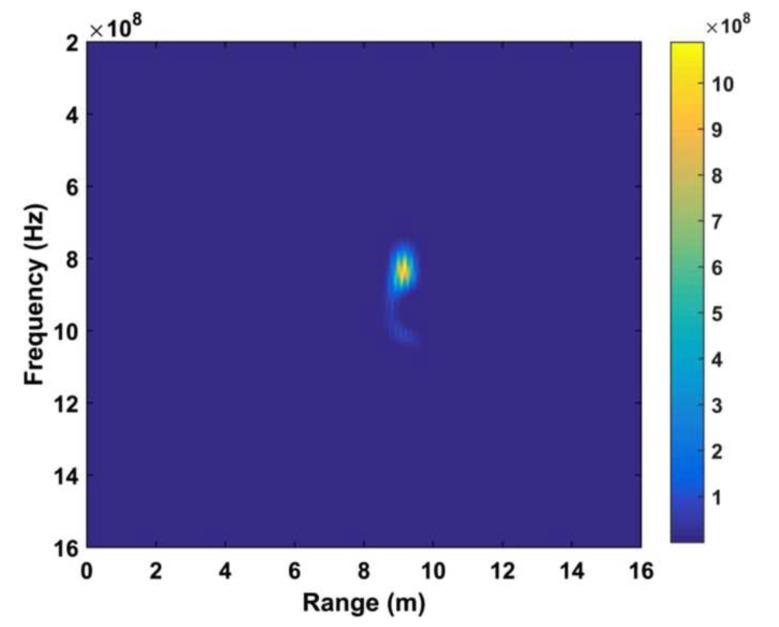
The STFT in the case of human location at a distance of 9 m [94].

Figure 21 displays the range information provided by this system when the distance from the person to the radar system was 9 m. The estimated range by their proposed system was quite accurate.

## 4. Conclusions and Future Perspectives

In this paper, some representative microwave Doppler sensor systems for finding survivors were discussed. For this special purpose, most of the considered systems operated at the *S* and *L* frequency bands. The power of the radar systems for this application was also much higher than the systems used for medical applications. Moreover, the signal processing techniques were very important in order to remove noise and clutter to improve the accuracy of the radar sensor systems.

In the future, optimized hardware structures can be combined with more efficient signal processing techniques to alleviate many of the issues with the radar vital sign-sensing system. Moreover, the Doppler sensor system may be combined with machine learning/deep learning techniques to enable more capabilities such as early warning of a heart attack or asthma attack. In addition, smarter and adaptive sensing systems for search and rescue purposes can be built based on AI. The data can be collected when the radar sensor operates in different environments. Then, these data can be used to build an AI model to enhance the accuracy and reduce the calibration complexity, which will lead to a smarter radar sensing system.

## Figures and Tables

**Table 1:** Parameters of the UWB radar [94].

Parameter	Value
Center frequency	400 MHz
Transmitted signal amplitude	50 V
Pulse repeat frequency (PRF)	600 kHz
Number of averaged values (NA)	30
Time window	124 ns
Number of samples (M)	4092
Input bandwidth of the analog to digital converter (ADC)	2.3 GHz
ADC sample size	12 bits
Receiver dynamic range	72 dB
